# Psychological distress among women with abnormal cervical cancer screening results in Poland: Validation of the Polish version of the Cervical Dysplasia Distress Questionnaire (CDDQ-PL)

**DOI:** 10.1371/journal.pone.0360240

**Published:** 2026-10-13

**Authors:** Agata Szmigiel-Soja, Justyna Kot, Klaudia Sieńko-Hans, Iwona Gawron, Robert Jach, Iwona Malinowska-Lipień, Dominika Trojnarska

**Affiliations:** 1 University Hospital in Kraków, Clinical Department of Gynecological Endocrinology and Gynecological Oncology, Kopernika, Kraków, Poland; 2 Jagiellonian University Medical College, Faculty of Health Sciences, Institute of Nursing and Midwifery, Department of Maternal and Child Health, Kopernika, Kraków, Poland; 3 Jagiellonian University Medical College, Faculty of Medicine, Chair of Gynecology and Obstetrics, Kopernika, Kraków, Poland; 4 Jagiellonian University Medical College, Faculty of Health Sciences, Institute of Nursing and Midwifery, Kopernika, Kraków, Poland; Teikyo University: Teikyo Daigaku, JAPAN

## Abstract

**Background:**

This study evaluated the validity and reliability of the Polish version of the Cervical Dysplasia Distress Questionnaire (CDDQ-PL) in women with abnormal cervical cancer screening results.

**Methods:**

A cross-sectional validation study was conducted among 285 women referred to a colposcopy unit following an abnormal cervical cancer screening result. Psychometric evaluation included language and content validity, item analysis, confirmatory factor analysis, internal consistency, and concurrent and convergent validity. Data were collected using a sociodemographic questionnaire, the CDDQ, an anxiety scale, and the Short Form Health Survey (SF-36).

**Results:**

The mean age of participants was 38.69 years (SD = 10.77). The mean total CDDQ-PL score was 2.21, with the highest distress observed for health consequences (3.02) and the lowest for embarrassment (1.47). Anxiety significantly decreased after the visit (6.51 ± 2.73 vs. 4.87 ± 2.95). Internal consistency was good, with Cronbach’s alpha coefficients ranging from 0.830 to 0.919 across subscales. Total CDDQ-PL scores showed moderate positive correlations with anxiety before (r = 0.378, p < 0.001) and after the visit (r = 0.381, p < 0.001). Higher distress was associated with poorer quality of life, particularly in emotional well-being, pain, and energy/fatigue domains of the SF-36.

**Conclusions:**

The Polish version of the CDDQ demonstrated satisfactory validity and good internal consistency for assessing psychological distress in women with abnormal cervical cancer screening results. Its temporal stability requires confirmation in future test–retest studies.

## Background

Cervical cancer prevention relies heavily on screening programs; accordingly, current guidelines in Poland recommend primary human papillomavirus (HPV) testing every 5 years with reflex cytology in HPV-positive cases, gradually replacing the traditional cytology-based screening performed every 3 years until mid-2026 [1]. Although screening programs have substantially reduced incidence and mortality, receiving information about an abnormal result has been consistently associated with significant psychological distress [2]. Anxiety experienced at this stage may adversely affect adherence to recommended diagnostic follow-up. It has been estimated that up to 60% of women who receive abnormal cervical cytology results do not proceed with further examinations, with emotional distress identified as an important contributing factor [3,4].

The psychosocial burden associated with abnormal screening results extends beyond immediate anxiety and may influence women’s perceptions of diagnostic procedures, health-related behaviors, and broader aspects of quality of life, including sexual functioning [5,6]. Therefore, accurate and standardized assessment of distress in this population is essential for both clinical practice and research. Such assessment enables the identification of women at higher risk of non-adherence and facilitates the development of targeted educational and psychological interventions aimed at improving follow-up rates and patient outcomes [7,8].

Given the lack of validated, culturally adapted tools for assessing distress associated with cervical dysplasia in the Polish population, this study aimed to evaluate the validity and reliability of the Polish version of the Cervical Dysplasia Distress Questionnaire (CDDQ-PL, Supporting Information S1 File CDDQ-PL with English version). Based on the obtained results, the instrument has been prepared for use in clinical and research settings in Poland, with the potential to inform future strategies for patient management and to improve care for women receiving abnormal cervical screening results.

The original CDDQ was developed as a condition-specific instrument designed to comprehensively evaluate anxiety related to cervical dysplasia and associated diagnostic processes [3]. It was based on the Psychosocial Effects of Abnormal Pap Smears Questionnaire (PEAPS-Q), a scale developed in 1995 by Bennetts et al. for measuring psychosocial distress experienced by women undergoing follow-up investigation after an abnormal Pap smear result and was validated by Shinn et al [3,9]. The questionnaire captures multiple dimensions of distress, including emotional responses to test results, concerns about medical procedures, and stress related to sexual and reproductive health. Its multidimensional structure allows for a nuanced understanding of patient experiences in the context of abnormal cervical findings. CDDQ validity and reliability studies have already been conducted in German, Serbian and Turkish [10–12]. The original scale was found to be valid and reliable, and it has been widely used and adapted to different samples.

The aim of this study was to translate and culturally adapt the CDDQ into Polish and to evaluate the validity and internal consistency of the resulting Polish version among women with abnormal cervical cancer screening results. To the best of our knowledge, this study constitutes the first attempt to adapt the scale into Polish, as confirmed by the original authors, who indicated that no prior permission for its translation and validation in the Polish language had been granted. No other valid or reliable instrument was found to adequately determine the stress status of individuals who had been admitted to the colposcopy unit. The CDDQ was evaluated as a suitable instrument for women in Poland in terms of its content, scope, and suitability.

## Methods

### Study design

This validation study was conducted using a cross-sectional research design.

### The convenience sample

Patients were referred to the University Hospital in Kraków, Poland, by their respective gynecologists following an abnormal cervical cancer screening result for further diagnostic evaluation and management. Women attending the colposcopy unit between September 2024 and September 2025 were eligible to participate if they were aged 18 years or older, had been referred following an abnormal cervical cancer screening result, provided written informed consent, and had sufficient proficiency in Polish to complete the study questionnaires.

The required sample size was estimated based on recommendations for psychometric analyses, suggesting inclusion of 5–10 participants per questionnaire item [13]. As the original scale consisted of 23 items, a minimum sample of 115–230 participants was considered adequate. Initially, 200 questionnaires were distributed. Owing to efficient recruitment and the subsequent inclusion of the SF-36 questionnaire, an additional 100 questionnaires were distributed.

### Data collection instruments

#### CDDQ.

Permission was obtained from the authors to adapt the CDDQ to Polish and to conduct a validity and reliability study.

The CDDQ scale consists of four subscales: tension and discomfort (6 items), embarrassment (2 items), sexual and reproductive consequences (9 items), and health consequences (6 items), as presented in Table 1. A total of 23 items is ranked on a 4-point Likert scale as 1 (Not at all), 2 (Somewhat), 3 (Moderately so), and 4 (Very much so). The overall score was obtained by taking the average of all item scores. The scale has no cutoff points, and higher scores indicate higher levels of stress.

**Table 1:** Original four-factor structure of the CDDQ.

Scale	Items
Tension and Discomfort	1, 2, 3, 4, 5, 6
Embarrassment	7, 8
Sexual and reproductive consequences	9, 10, 11, 12, 13, 14, 15, 16, 17
Health consequences	18, 19, 20, 21, 22, 23

#### Anxiety questionnaire.

Anxiety was assessed using a structured, self-administered questionnaire. The questionnaire included items evaluating anxiety levels before and after the colposcopy visit using a 10-point scale, as well as questions on characteristics. The instrument was developed based on a review of the literature and clinical experience and was pilot-tested prior to the study.

The researcher-developed sociodemographic part of the questionnaire comprised four items assessing age, place of residence, educational level, and marital status.

#### SF-36.

During the course of the study, the research team incorporated the Short Form Health Survey questionnaire (SF-36), a widely used generic instrument for assessing health-related quality of life, to enable a more comprehensive evaluation and to support the validation process of the CDDQ-PL. The inclusion of this measure allowed for the examination of convergent validity between distress related to cervical dysplasia and broader quality-of-life domains.

The SF-36 is a very popular instrument for evaluating health-related quality of life [14,15]. The SF-36 measures eight scales: physical functioning, role limitations due to physical health, role limitations due to emotional problems, energy/ fatigue, emotional well-being, social functioning, pain, general health. Prior to the commencement of the study, formal authorization to use the SF-36 questionnaire was obtained from the Medical Outcomes Study (MOS) and the Medical Outcomes Trust (MOT), which granted permission for its use in academic research. The validated Polish version of the SF-36 was applied in the present study [16]. Scoring of the questionnaires was performed in accordance with the RAND MOS scoring algorithm, using the instrument and guidelines available online [17].

#### Polish adaptation process of the CDDQ.

The CDDQ was translated and culturally adapted into Polish in accordance with the five-stage model proposed by Brislin, Lonner, and Thorndike: forward translation into the target language, evaluation of the initial translation, back-translation into the source language, comparison of the back-translated and original versions, and final expert evaluation [18,19]. Forward translation was performed independently by a linguist and a professional translator. After the initial translations had been reviewed and reconciled, the Polish version was back-translated into English by an independent bilingual translator fluent in Polish and English. The back-translated version was compared with the original questionnaire to identify and resolve any discrepancies.

Throughout the adaptation process, particular attention was given to semantic, conceptual, and cultural equivalence to preserve the meaning and clinical relevance of the original items. The translated version was evaluated by a panel of five experts comprising two psychologists, one gynecologist, and two gynecologic oncologists. The experts assessed the items for linguistic clarity, conceptual equivalence, cultural appropriateness, and clinical relevance, in accordance with established standards [20]. The final wording was agreed upon by consensus. Content validity was evaluated qualitatively; a quantitative content validity index was not calculated.

Formal cognitive debriefing was subsequently conducted with 10 Polish patients from the target population. After completing the questionnaire, participants were interviewed about the clarity and comprehensibility of the items, the suitability of the response options, and the cultural relevance of the content. Their feedback was considered when finalizing the CDDQ-PL.

### Data collection

The purpose and content of the study were explained to all patients who met the inclusion criteria. During their initial visit, they were given the questionnaires. Participants completed the questionnaires after the examination, without interference from medical staff. No personally identifiable information was collected. Before completing the questionnaires, participants were informed that they could ask questions or request clarification at any time. After returning the completed forms, each participant was offered a further opportunity to speak with the physician and was asked whether any questionnaire item had been unclear or difficult to understand. This procedure was used to identify potential problems with the comprehensibility of the translated items. Participant feedback did not indicate recurring difficulties related to the wording or cultural interpretation of the CDDQ-PL.

A total of 300 questionnaires were distributed, including 100 that contained the SF-36 instrument. Of these, 294 questionnaires were returned, including 98 with the SF-36. Nine questionnaires were excluded due to incomplete data, of which 3 included the SF-36. Finally, 285 questionnaires were included in the final analysis, including 95 with completed SF-36 data (Fig 1).

**Fig 1:**
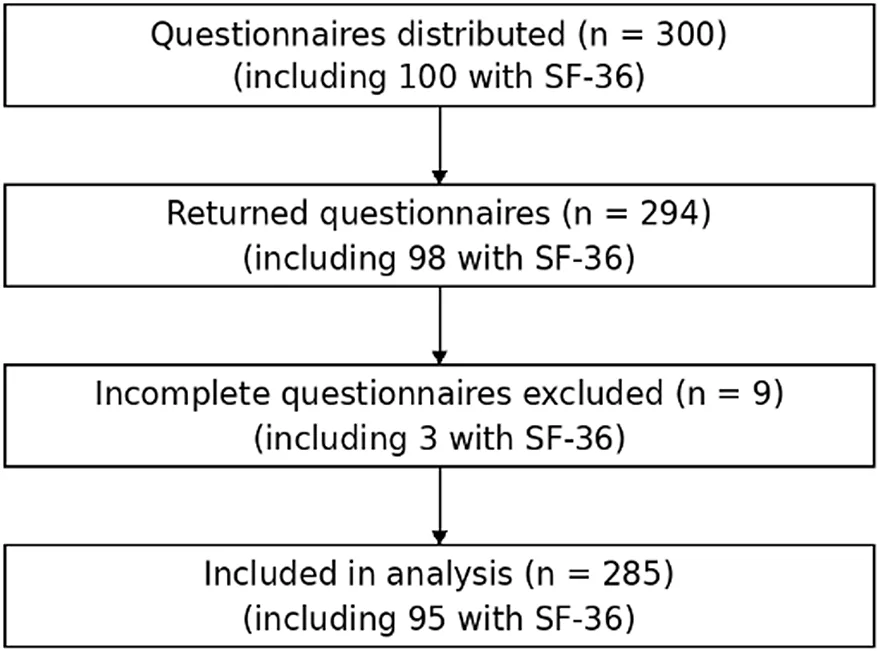
Study flow diagram of questionnaire distribution and inclusion.

### Statistical methods

Descriptive statistics were used to summarize the data. Continuous variables are presented as means with standard deviations (SD) or medians with interquartile range (IQR), as appropriate, while categorical variables are reported as counts and percentages.

Changes in anxiety levels before and after the visit were assessed using paired comparisons.

Psychometric evaluation of the CDDQ-PL included item analysis, reliability assessment, and validity testing. Item-level analysis comprised evaluation of floor and ceiling effects, defined as the proportion of respondents selecting the lowest and highest possible response options, respectively.

Because the CDDQ is an established instrument with a predefined subscale structure, exploratory factor analysis (EFA) was not conducted. Construct validity of the CDDQ was assessed using confirmatory factor analysis (CFA) based on the original four-factor structure. Given the ordinal nature of the data, the weighted least squares mean, and variance adjusted (WLSMV) estimator was applied because responses were ordinal Likert-type variables. Model fit was evaluated using the chi-square to degrees of freedom ratio (χ²/df), comparative fit index (CFI), root mean square error of approximation (RMSEA), and standardized root mean square residual (SRMR). CFI ≥ 0.90, RMSEA ≤0.08, and SRMR ≤0.08 were considered indicative of acceptable fit, whereas CFI ≥ 0.95, RMSEA ≤0.06, and SRMR ≤0.08 represented more stringent criteria. The two-index strategy proposed by Hu and Bentler was also applied; according to this approach, model fit is supported when SRMR is < 0.09 and either CFI is > 0.96 or RMSEA is < 0.06.

Floor and ceiling effects were calculated as the percentages of participants selecting the lowest and highest possible response categories, respectively. An effect was considered present when more than 15% of participants selected the corresponding extreme category and was described as pronounced when this proportion was at least 50%.

Internal consistency was assessed using Cronbach’s alpha coefficients, with values ≥0.70 considered acceptable. Item-total correlations and changes in alpha following item removal were also examined.

Construct validity was further evaluated using Spearman’s rank-order correlation coefficients. Convergent validity was assessed by examining correlations between the CDDQ-PL total and subscale scores and anxiety scores before and after the visit. Correlations between the CDDQ-PL scores and individual SF-36 domains were examined to assess the expected relationships between disease-specific distress and health-related quality of life.

Statistical significance was set at p < 0.05.

All analyses were conducted using R software, version 4.5.3 (R Foundation for Statistical Computing, Vienna, Austria), with the lavaan package (Yves Rosseel, Ghent University, Ghent, Belgium).

### Ethics approval and consent to participate

This study was conducted in accordance with the principles of the Declaration of Helsinki and received ethical approval from the Jagiellonian University Medical College Research Ethics Committee (Approval No. 118.0043.1.280.2024, dated 26.09.2024; S2 File Human Participants Research Checklist). No ethical concerns were raised regarding the conduct of the study. Written informed consent was obtained from each participant.

## Results

### Sociodemographic characteristics

A total of 285 women were included in the analysis. The mean age was 38.69 years (SD 10.77). Most participants lived in cities with >500,000 inhabitants (57.54%), while 23.86% resided in rural areas. The majority had higher education (60.35%), and 46.67% were married. The detailed results are presented in Table 2.

**Table 2:** Sociodemographic characteristics of respondents (N = 285).

Age [years]	
Mean (SD)	38.69 (10.77)
Median (quartiles)	37 (31-45)
Range	18-71
**Place of residence**	n (%)
City with >500,000 inhabitants	164 (57.54)
City with 250,000-500,000 inhabitants	15 (5.26)
City with 100,000-250,000 inhabitants	11 (3.86)
City with <100,000 inhabitants	26 (9.12)
Rural area	68 (23.86)
Unknown	1 (0.35)
**Education**	n (%)
Higher (tertiary)	172 (60.35)
Secondary	94 (32.98)
Basic vocational	14 (4.91)
Lower secondary (middle school)	3 (1.05)
Primary	2 (0.70)
**Marital status**	n (%)
Single	107 (37.54)
Married	133 (46.67)
Divorced	35 (12.28)
Widowed	10 (3.51)

### CDDQ-PL scores

The mean total CDDQ-PL score was 2.21. The highest subscale score was observed for health consequences (3.02), while embarrassment was lowest (1.47). The results are summarized in Table 3.

**Table 3:** Results of the CDDQ-PL (N = 285).

Item	Mean	SD	Median	Min	Max	Q1	Q3
Total Score	2.21	0.46	2.17	1	3.48	1.91	2.52
Tension and Discomfort	1.96	0.59	2	1	3.67	1.5	2.4
Embarrassment	1.47	0.58	1	1	3.5	1	2
Sexual and reproductive consequences	2	0.6	2	1	3.67	1.56	2.33
Health consequences	3.02	0.69	3	1	4	2.5	3.67

### Anxiety levels before and after the visit

As illustrated in Table 4, mean anxiety decreased from 6.51 (SD 2.73) before the visit to 4.87 (SD 2.95) after the visit.

**Table 4:** Results of the anxiety questionnaire (N = 285).

Anxiety before the visit	
Mean (SD)	6.51 (2.73)
Median (quartiles)	7 (5 [–]9)
Range	1-10
**Anxiety after the visit**	
Mean (SD)	4.87 (2.95)
Median (quartiles)	5 (2 [–]8)
Range	1-10

### SF-36 quality of life

SF-36 scores were highest for physical functioning (94.68%) and role limitations due to physical health (83.42%), and lowest for general health (49.37%) and energy/fatigue (55.32%). The results are displayed in Table 5.

**Table 5:** Results of the SF-36 questionnaire (N = 95).

Parameter	Mean	SD	Median	Min	Max	Q1	Q3
Physical functioning (%)	94.68	10.05	100	50	100	95	100
Role limitations due to physical health (%)	83.42	30.64	100	0	100	75	100
Role limitations due to emotional problems (%)	83.16	32.21	100	0	100	83.34	100
Energy/ fatigue (%)	55.32	16.48	55	15	90	45	65
Emotional well-being (%)	66.11	16.08	68	20	92	56	76
Social functioning (%)	74.47	23.91	75	12.5	100	56.25	100
Pain (%)	72.68	25.6	80	0	100	65	90
General health (%)	49.37	10.29	50	15	75	40	55

### Item analysis

Floor effects were substantial for several items, exceeding 50% for items 2, 7, and 8, while ceiling effects were generally low but reached over 30% for items 18–20. Detailed data are provided in Table 6.

**Table 6:** Item analysis of CDDQ.

Item	Floor effect	Ceiling effect	Missing
Item 1	39.3%	4.2%	0.4%
Item 2	50.5%	0.7%	0.0%
Item 3	29.5%	3.5%	0.0%
Item 4	39.3%	1.4%	0.0%
Item 5	18.6%	5.6%	0.0%
Item 6	21.4%	5.6%	0.0%
Item 7	57.5%	1.1%	0.0%
Item 8	61.8%	0.0%	0.0%
Item 9	44.6%	17.5%	6.7%
Item 10	19.3%	18.6%	0.4%
Item 11	29.8%	11.6%	0.4%
Item 12	31.2%	2.5%	0.7%
Item 13	41.8%	3.9%	0.4%
Item 14	28.8%	3.5%	0.4%
Item 15	44.6%	6.0%	0.4%
Item 16	30.9%	1.4%	0.7%
Item 17	31.6%	3.5%	0.4%
Item 18	2.1%	42.1%	0.4%
Item 19	2.1%	32.6%	0.0%
Item 20	1.1%	33.7%	0.0%
Item 21	11.2%	22.5%	0.0%
Item 22	0.7%	35.4%	0.4%
Item 23	6.3%	27.4%	0.0%

### Internal consistency

The CDDQ-PL demonstrated good internal consistency. Cronbach’s alpha coefficients were 0.830 for the tension and discomfort subscale, 0.845 for sexual and reproductive consequences, and 0.919 for health consequences. The details are shown in Table 7.

**Table 7:** Cronbach’s alpha coefficients for individual CDDQ-PL subscales.

Scale	Cronbach’s alpha
Tension and Discomfort	0.830
Sexual and reproductive consequences	0.845
Health consequences	0.919

As presented in Table 8, item-total correlations were generally acceptable, although lower values were observed for some items (e.g., item 9).

**Table 8:** Item–total correlations for the CDDQ-PL.

Scale	Item	Alpha when item omitted	Item-total correlation
Tension and Discomfort	Item 1	0.818	0.527
	Item 2	0.810	0.568
	Item 3	0.781	0.700
	Item 4	0.830	0.455
	Item 5	0.775	0.729
	Item 6	0.796	0.635
Sexual and reproductive consequences	Item 9	0.863	0.319
	Item 10	0.832	0.533
	Item 11	0.816	0.666
	Item 12	0.822	0.639
	Item 13	0.814	0.699
	Item 14	0.818	0.669
	Item 15	0.823	0.608
	Item 16	0.834	0.511
	Item 17	0.831	0.543
Health consequences	Item 18	0.904	0.787
	Item 19	0.914	0.700
	Item 20	0.897	0.829
	Item 21	0.912	0.734
	Item 22	0.901	0.795
	Item 23	0.901	0.799

### Correlations with anxiety

CDDQ-PL scores were positively correlated with anxiety levels both before and after the visit. The total CDDQ-PL score showed moderate positive correlations with anxiety before (r = 0.378, p < 0.001) and after the visit (r = 0.381, p < 0.001). The health consequences subscale showed the strongest correlations with anxiety (r = 0.48 before and r = 0.434 after the visit; both p < 0.001). Correlations for the tension and discomfort and sexual and reproductive consequences subscales were weaker (r = 0.167–0.261), while embarrassment was not significantly correlated with anxiety. Results are detailed in Table 9.

**Table 9:** Correlations between CDDQ-PL scores and anxiety levels before and after the visit.

Anxiety	CDDQ-PL Total	Tension and Discomfort	Embarrassment	Sexual and reproductive consequences	Health consequences
Anxiety before the visit	r = 0.378 p < 0.001 *	r = 0.167 p=0.005 *	r = 0.105 p = 0.076	r = 0.228 p < 0.001 *	r = 0.48 p < 0.001 *
Anxiety after the visit	r = 0.381 p < 0.001 *	r = 0.172 p=0.004 *	r = 0.099 p = 0.096	r = 0.261 p < 0.001 *	r = 0.434 p < 0.001 *

### Correlations with quality of life (SF-36)

Higher CDDQ-PL scores were associated with poorer quality of life. Significant negative correlations were observed between the total CDDQ-PL score and several SF-36 domains, including role limitations due to emotional problems (r = −0.32, p = 0.002), energy/fatigue (r = −0.262, p = 0.01), emotional well-being (r = −0.314, p = 0.002), social functioning (r = −0.242, p = 0.018), pain (r = −0.379, p < 0.001), and general health (r = −0.242, p = 0.018). Similar patterns were observed across subscales, particularly for health consequences, which showed strong associations with emotional well-being and energy levels. The correlations are presented in Table 10.

**Table 10:** Correlations between CDDQ-PL scores and SF-36.

SF-36	CDDQ-PL Total	Tension and Discomfort	Embarrassment	Sexual and reproductive consequences	Health consequences
Physical functioning (%)	r = −0.002 p = 0.988	r = 0.037 p = 0.718	r = 0.01 p = 0.925	r = 0.104 p = 0.317	r = −0.126 p = 0.223
Role limitations due to physical health (%)	r = −0.14 p = 0.177	r = −0.239 p=0.02 *	r = −0.158 p = 0.127	r = −0.081 p = 0.435	r = 0 p = 1
Role limitations due to emotional problems (%)	r = −0.32 p=0.002 *	r = −0.201 p = 0.051	r = −0.194 p = 0.059	r = −0.234 p=0.022 *	r = −0.23 p=0.025 *
Energy/ fatigue (%)	r = −0.262 p=0.01 *	r = −0.107 p = 0.302	r = 0.018 p = 0.863	r = −0.197 p = 0.055	r = −0.242 p=0.018 *
Emotional well-being (%)	r = −0.314 p=0.002 *	r = −0.173 p = 0.093	r = −0.115 p = 0.267	r = −0.129 p = 0.212	r = −0.371 p < 0.001 *
Social functioning (%)	r = −0.242 p=0.018 *	r = −0.163 p = 0.114	r = −0.265 p=0.009 *	r = −0.213 p=0.038 *	r = −0.116 p = 0.265
Pain (%)	r = −0.379 p < 0.001 *	r = −0.321 p=0.002 *	r = −0.277 p=0.007 *	r = −0.269 p=0.008 *	r = −0.269 p=0.008 *
General health (%)	r = −0.242 p=0.018 *	r = −0.322 p=0.001 *	r = −0.104 p = 0.315	r = −0.155 p = 0.133	r = −0.138 p = 0.181

## Discussion

This study evaluated the validity and reliability of the Polish version of the CDDQ (CDDQ-PL) and confirmed its suitability for assessing disease-specific distress among women with abnormal cervical cancer screening results. The findings demonstrate that the CDDQ-PL is a robust tool for measuring emotional and cognitive responses to abnormal cytology, consistent with previous adaptations of the instrument in Turkey, Serbia, and Germany [10–12].

No normative data have been established for the CDDQ; therefore, raw scores cannot be classified as high or low levels of distress. The total and subscale scores range from 1 to 4, with higher scores indicating higher distress levels. In this study, the highest distress was related to health consequences (mean = 3.02) and sexual and reproductive consequences (mean = 2.00), while embarrassment (mean = 1.47) and tension and discomfort (mean = 1.96) were the lowest. These findings align with prior studies showing that health-related concerns typically predominate among women confronted with abnormal cervical screening results, reflecting fears about diagnosis, treatment, and cancer outcomes [3].

Item-level analysis revealed notable floor effects for several items, particularly items 2, 7, and 8, suggesting limited variability in responses. This pattern may indicate that Polish women are relatively familiar with gynecological examinations and experience less embarrassment in this context compared to previously studied populations. Similar findings were reported in the Turkish validation of the CDDQ, where cultural familiarity with medical procedures influenced specific item performance [12].

Internal consistency was high across all subscales, exceeding the recommended threshold of 0.70 and confirming that all items measure a coherent construct of distress [21]. All item–total correlations were positive, indicating that the CDDQ-PL items contributed meaningfully to their domains. This reliability profile parallels those obtained in other language versions, supporting the scale’s cross-cultural stability. [11,12]

Confirmatory factor analysis supported the original four-factor structure of the instrument. According to the two-index strategy proposed by Hu and Bentler, the observed fit indices meet acceptable criteria, reinforcing the adequacy of the theoretical model. [22] All factor loadings were statistically significant, confirming the distinctiveness and internal coherence of each dimension of the CDDQ-PL. However, given that CFA is sensitive to sample size, future studies should consider replication on larger datasets to further verify structural robustness.

An important limitation of this study is that test–retest reliability was not assessed. The cross-sectional design involved a single administration of the CDDQ-PL and therefore allowed the evaluation of internal consistency but not the temporal stability or reproducibility of the scores. Consequently, the high Cronbach’s alpha coefficients should not be interpreted as evidence that the instrument produces stable results over time. This limitation restricts the conclusions that can be drawn regarding the overall reliability of the CDDQ-PL. Prospective studies in which the questionnaire is administered repeatedly using an appropriate retest interval are needed to estimate test–retest reliability and determine whether changes in scores reflect genuine changes in distress rather than measurement variability.

Regarding content validity, the Polish version underwent rigorous translation and back-translation following the Brislin model, ensuring linguistic and conceptual equivalence. [18] Cultural considerations are crucial across validation processes, particularly in sensitive health domains such as gynecologic oncology. Nonetheless, the psychosocial stressors linked to cervical dysplasia appear consistent across different cultures – dominated by uncertainty, perceived cancer risk, and concerns about reproductive health- thus supporting the conceptual universality of the CDDQ. [23]

External validity was demonstrated by cross-study comparability: the mean scores and subscale structure were analogous to results observed in other European populations. [12] This consistency suggests that the underlying construct of health-related distress following abnormal cytology has a common psychosocial basis.

Construct validity was supported by two complementary findings. First, moderate to strong positive correlations between CDDQ-PL and anxiety levels both before and after colposcopy support its convergent validity. Second, negative correlations with SF-36 domains – particularly vitality, emotional well-being, and social functioning – confirm expected discriminant relationships. These patterns are consistent with literature showing that psychological distress adversely affects quality-of-life outcomes among women undergoing cervical evaluation. [22] Moreover, moderate correlations rather than excessively high ones indicate that the CDDQ-PL captures disease-specific psychological impacts beyond general anxiety or quality-of-life measures.

Criterion-related validity was not assessed in the present study. Concurrent criterion validity could not be evaluated because no validated Polish condition-specific instrument or accepted diagnostic gold standard was available against which CDDQ-PL scores could be compared. In addition, the cross-sectional design did not include subsequent clinical or behavioral outcomes and therefore did not allow predictive validity to be examined. Known-group validity was also not evaluated because clinically distinct groups expected a priori to differ in distress were not prospectively defined. Consequently, the present findings support the construct validity of the CDDQ-PL but do not establish its ability to distinguish between clinically relevant groups or predict subsequent outcomes. Future prospective studies should investigate whether baseline CDDQ-PL scores predict attendance at recommended follow-up examinations, adherence to diagnostic or therapeutic procedures, treatment engagement, or the need for psychological support, which are critically important in cervical cancer prevention. [24] Known-group validity could be examined by comparing prespecified groups defined according to factors such as the severity of the screening abnormality, histopathological diagnosis, previous experience of abnormal screening results, or the need for further treatment. Incorporating such patient reported measures into routine care could enhance psychosocial support, improve compliance with diagnostic procedures, and ultimately strengthen cervical cancer prevention efforts in Poland, where health literacy and preventive participation remain uneven. [25] Providing psychological support could lead to increased participation in public screening programs, which would enable the improvement of cervical cancer prevention and treatment initiatives, addressing the currently unsatisfactory outcomes. [26,27]

The CDDQ-PL may have practical applications at several stages of the cervical screening pathway. It could be administered after a woman is informed of an abnormal screening result or before colposcopy as a brief, structured assessment of concerns related to the examination, embarrassment, sexual and reproductive consequences, and possible health outcomes. Reviewing individual responses may help healthcare professionals identify the sources of distress most relevant to a particular patient, address misconceptions, tailor the information provided, and improve communication before diagnostic procedures. Women reporting substantial distress could receive more detailed counselling, additional educational materials, or referral for psychological support when clinically appropriate.

The questionnaire may also have future value in identifying women at increased risk of non-attendance or disengagement from diagnostic follow-up. Nevertheless, the present study did not assess predictive validity and the CDDQ has no established clinical cutoff scores. The CDDQ-PL should therefore not currently be used as a standalone tool to predict non-adherence or determine referral for psychological care. Prospective studies should examine whether baseline scores are associated with attendance at colposcopy, completion of recommended diagnostic and therapeutic procedures, and the subsequent need for psychological support. Establishing clinically meaningful thresholds would be necessary before the instrument could be incorporated into formal screening or referral pathways.

This study has several limitations. First, although the sample size met commonly accepted recommendations for psychometric analyses, a larger sample would provide more robust and stable estimates in confirmatory factor analysis. Replication in larger independent samples is therefore needed to confirm the four-factor structure of the CDDQ-PL.

Second, participants were recruited through convenience sampling at a single tertiary colposcopy center, which may have introduced selection bias and limits the generalizability of the findings. Women referred to and attending a university-based specialist center may differ from those undergoing screening or follow-up in primary care in terms of clinical characteristics, previous screening experiences, access to healthcare, health literacy, and psychological distress. Furthermore, only women who attended their referral appointment and agreed to participate were included. Women who did not attend follow-up—potentially because of anxiety, socioeconomic barriers, or limited access to care—may therefore be underrepresented.

Third, the sample was predominantly urban and highly educated, with most participants having higher education and living in a city with more than 500,000 inhabitants. Educational attainment, socioeconomic circumstances, health literacy, and place of residence may influence the understanding of abnormal screening results, perceived cancer risk, expression of distress, and interpretation of questionnaire items. The distribution of CDDQ-PL scores and its psychometric performance may consequently differ among women with lower educational attainment or health literacy and among those living in rural or underserved areas. The present findings should therefore not be generalized uncritically to the entire Polish screening population. Future validation studies should use multicenter recruitment encompassing tertiary centers, primary care practices, and community screening programs across diverse geographical and socioeconomic settings. Larger and more heterogeneous samples would also permit assessment of measurement invariance and differential item functioning across educational, socioeconomic, and residential groups.

Nine of 294 returned questionnaires (3.1%) were excluded because of incomplete data. Although participants could request clarification and reported no recurring difficulties with the translated items, reasons for individual missing responses were not systematically recorded. Future studies should document these reasons and use cognitive interviews to further assess item comprehension and cultural relevance.

An additional limitation concerns the assessment of associations between the CDDQ-PL and health-related quality of life. Because the SF-36 was introduced later during recruitment, it was completed by only 95 of the 285 participants. Consequently, analyses involving SF-36 domains were based on approximately one-third of the total sample. The smaller subsample reduced statistical power, increased the uncertainty of the correlation estimates, and may have limited the detection of weaker associations. Moreover, although inclusion in this subsample was determined by the timing of recruitment rather than by participants’ clinical characteristics, potential systematic differences between women recruited before and after the introduction of the SF-36 cannot be excluded. Selection bias may therefore have affected the observed validity estimates. Accordingly, the associations between the CDDQ-PL and SF-36 domains should be interpreted cautiously and considered preliminary. Future studies should administer both instruments to the entire study population and confirm these relationships in larger, more representative samples.

## Conclusions

The Polish adaptation of the CDDQ demonstrated good internal consistency and satisfactory evidence of content and construct validity, including support for its four-factor structure. These findings indicate that the CDDQ-PL is a promising instrument for assessing distress among women with abnormal cervical cancer screening results. However, because test–retest reliability was not evaluated, no conclusions can yet be drawn regarding the temporal stability or reproducibility of its scores. Further prospective studies incorporating repeated administration of the questionnaire are required before the overall reliability of the CDDQ-PL can be considered fully established.

## Supporting information

S1 File English version of CDDQ. (PDF)

S2 File Human Participants Research Checklist. (PDF)

## Abbreviations

CDDQ: Cervical Dysplasia Distress Questionnaire
CDDQ-PL: Polish version of the Cervical Dysplasia Distress Questionnaire
CFA: confirmatory factor analysis
CFI: comparative fit index
CI: confidence intervals
EFA: exploratory factor analysis
HPV: human papillomavirus
IQR: interquartile range
MOS: Medical Outcomes Study
MOT: Medical Outcomes Trust
OR: odds ratios
PEAPS-Q: Psychosocial Effects of Abnormal Pap Smears Questionnaire
Q1: lower quartile
Q3: upper quartile
RMSEA: root mean square error of approximation
SD: standard deviation
SF-36: Short Form Health Survey questionnaire
SRMR: standardized root mean square residual
WLSMV: weighted least squares mean, and variance adjusted
χ^2^/df: chi-square to degrees of freedom ratio
