# Supplementary material for: Psychological distress among women with abnormal cervical cancer screening results in Poland: Validation of the Polish version of the Cervical Dysplasia Distress Questionnaire (CDDQ-PL)
Source: PLoS One. 2026 Oct 13;21(10):e0360240. doi: 10.1371/journal.pone.0360240 (PMC13660920; doi:10.1371/journal.pone.0360240)
Supplement: Supplementary file 1 [file pone.0360240.s001.pdf]

CDDQ

1. Did you find the exams uncomfortable?

☐ Not at all. ☐ Somewhat. ☐ Moderately so. ☐ Very much so.

2. Did you find the exams emotionally upsetting?

☐ Not at all. ☐ Somewhat. ☐ Moderately so. ☐ Very much so.

3. Did the exams make you nervous?

☐ Not at all. ☐ Somewhat. ☐ Moderately so. ☐ Very much so.

4. Did the exam hurt?

☐ Not at all. ☐ Somewhat. ☐ Moderately so. ☐ Very much so.

5. Did you feel tense?

☐ Not at all. ☐ Somewhat. ☐ Moderately so. ☐ Very much so.

6. Were you nervous?

☐ Not at all. ☐ Somewhat. ☐ Moderately so. ☐ Very much so.

7. Were you uncomfortable being partly undressed?

☐ Not at all. ☐ Somewhat. ☐ Moderately so. ☐ Very much so.

8. Were you embarrassed having your private parts touched by the doctors or the nurse?

☐ Not at all. ☐ Somewhat. ☐ Moderately so. ☐ Very much so.

9. How worried are you that you would lose your chance to have a baby?

☐ Not at all. ☐ Somewhat. ☐ Moderately so. ☐ Very much so.

10. Have you been worried that you could give the problem to a sexual partner?

☐ Not at all. ☐ Somewhat. ☐ Moderately so. ☐ Very much so.

11. Have you been worried whether a sexual partner will think they can catch the problems from you?

☐ Not at all. ☐ Somewhat. ☐ Moderately so. ☐ Very much so.

12. Have you been worried whether you should continue having sex?

□ Not at all. □ Somewhat. □ Moderately so. □ Very much so.

13. Have you been worried that this problem might affect how attractive you are to your sexual partner?

□ Not at all. □ Somewhat. □ Moderately so. □ Very much so.

14. Have you been worried whether having sex will make the problem worse?

□ Not at all. □ Somewhat. □ Moderately so. □ Very much so.

15. Have you been worried whether others think you have had more sexual partners than you should?

□ Not at all. □ Somewhat. □ Moderately so. □ Very much so.

16. Have you been worried about sex being more painful now?

□ Not at all. □ Somewhat. □ Moderately so. □ Very much so.

17. Have you been worried that this problem might affect how much you enjoy sex?

□ Not at all. □ Somewhat. □ Moderately so. □ Very much so.

18. How worried are you that cancer will appear in your body?

□ Not at all. □ Somewhat. □ Moderately so. □ Very much so.

19. Have you worried about the test results?

□ Not at all. □ Somewhat. □ Moderately so. □ Very much so.

20. Have you worried that you may have cancer?

□ Not at all. □ Somewhat. □ Moderately so. □ Very much so.

21. How worried are you that you might die?

□ Not at all. □ Somewhat. □ Moderately so. □ Very much so.

22. Have you been worried that your problem may turn into cancer?

□ Not at all. □ Somewhat. □ Moderately so. □ Very much so.

23. How worried are you that you might die from cervical cancer?

□ Not at all. □ Somewhat. □ Moderately so. □ Very much so.
