# Supplementary material for: Psychological distress among women with abnormal cervical cancer screening results in Poland: Validation of the Polish version of the Cervical Dysplasia Distress Questionnaire (CDDQ-PL)
Source: PLoS One. 2026 Oct 13;21(10):e0360240. doi: 10.1371/journal.pone.0360240 (PMC13660920; doi:10.1371/journal.pone.0360240)
Supplement: Peer review history

# Peer review: Decision Letter 0

**DOI:** 10.1371/journal.pone.0360240.r001

**Authors:** Kazunori Nagasaka

29 Jul 2026

Dear Dr. Trojnarska,

Thank you for submitting your manuscript to PLOS ONE. After careful consideration, we feel that it has merit but does not fully meet PLOS ONE’s publication criteria as it currently stands. Therefore, we invite you to submit a revised version of the manuscript that addresses the points raised during the review process.

publication criteria and not, for example, on novelty

Please submit your revised manuscript by Aug 28 2026 11:59PM. If you will need more time than this to complete your revisions, please reply to this message or contact the journal office at plosone@plos.org. When you're ready to submit your revision, log on to https://www.editorialmanager.com/pone/ and select the 'Submissions Needing Revision' folder to locate your manuscript file.

- A letter that responds to each point raised by the academic editor and reviewer(s). You should upload this letter as a separate file labeled 'Response to Reviewers'.
- A marked-up copy of your manuscript that highlights changes made to the original version. You should upload this as a separate file labeled 'Revised Manuscript with Track Changes'.
- An unmarked version of your revised paper without tracked changes. You should upload this as a separate file labeled 'Manuscript'.

If you would like to make changes to your financial disclosure, please include your updated statement in your cover letter. Guidelines for resubmitting your figure files are available below the reviewer comments at the end of this letter.

If applicable, we recommend that you deposit your laboratory protocols in protocols.io to enhance the reproducibility of your results. Protocols.io assigns your protocol its own identifier (DOI) so that it can be cited independently in the future. For instructions see: https://journals.plos.org/plosone/s/submission-guidelines#loc-laboratory-protocols. Additionally, PLOS ONE offers an option for publishing peer-reviewed Lab Protocol articles, which describe protocols hosted on protocols.io. Read more information on sharing protocols at https://plos.org/protocols?utm_medium=editorial-email&utm_source=authorletters&utm_campaign=protocols.

As the corresponding author, your ORCID iD is verified in the submission system and will appear in the published article. PLOS supports the use of ORCID, and we encourage all coauthors to register for an ORCID iD and use it as well. Please encourage your coauthors to verify their ORCID iD within the submission system before final acceptance, as unverified ORCID iDs will not appear in the published article. *Only*  the individual author can complete the verification step; PLOS staff *cannot*  verify ORCID iDs on behalf of authors.

We look forward to receiving your revised manuscript.

Kind regards,

Kazunori Nagasaka

Academic Editor

PLOS One

**Journal Requirements:**

When submitting your revision, we need you to address these additional requirements.

1. Please ensure that your manuscript meets PLOS ONE's style requirements, including those for file naming. The PLOS ONE style templates can be found at

https://journals.plos.org/plosone/s/file?id=wjVg/PLOSOne_formatting_sample_main_body.pdf and

https://journals.plos.org/plosone/s/file?id=ba62/PLOSOne_formatting_sample_title_authors_affiliations.pdf

2. Thank you for stating the following in the Acknowledgments Section of your manuscript:

“The article processing charge was funded by internal funds of the Jagiellonian University Medical College. The authors would like to thank all colleagues working at the colposcopy unit of the University Hospital in Kraków for their assistance in the dissemination of questionnaires and support during the study.”

We note that you have provided funding information that is not currently declared in your Funding Statement. However, funding information should not appear in the Acknowledgments section or other areas of your manuscript. We will only publish funding information present in the Funding Statement section of the online submission form.

Please remove any funding-related text from the manuscript and let us know how you would like to update your Funding Statement. Currently, your Funding Statement reads as follows:

“The author(s) received no specific funding for this work.”

Please include your amended statements within your cover letter; we will change the online submission form on your behalf.

3. In the online submission form you indicate that your data is not available for proprietary reasons and have provided a contact point for accessing this data. Please note that your current contact point is a co-author on this manuscript. According to our Data Policy, the contact point must not be an author on the manuscript and must be an institutional contact, ideally not an individual. Please revise your data statement to a non-author institutional point of contact, such as a data access or ethics committee, and send this to us via return email. Please also include contact information for the third party organization, and please include the full citation of where the data can be found.

4. Your ethics statement should only appear in the Methods section of your manuscript. If your ethics statement is written in any section besides the Methods, please move it to the Methods section and delete it from any other section. Please ensure that your ethics statement is included in your manuscript, as the ethics statement entered into the online submission form will not be published alongside your manuscript.

5. Please remove your figures from within your manuscript file, leaving only the individual TIFF/EPS image files, uploaded separately. These will be automatically included in the reviewers’ PDF.

6. Please include captions for your Supporting Information files at the end of your manuscript, and update any in-text citations to match accordingly. Please see our Supporting Information guidelines for more information: http://journals.plos.org/plosone/s/supporting-information.

7. If the reviewer comments include a recommendation to cite specific previously published works, please review and evaluate these publications to determine whether they are relevant and should be cited. There is no requirement to cite these works unless the editor has indicated otherwise.

8. Please review your reference list to ensure that it is complete and correct. If you have cited papers that have been retracted, please include the rationale for doing so in the manuscript text, or remove these references and replace them with relevant current references. Any changes to the reference list should be mentioned in the rebuttal letter that accompanies your revised manuscript. If you need to cite a retracted article, indicate the article’s retracted status in the References list and also include a citation and full reference for the retraction notice.

**Additional Editor Comments:**

Dear Author,

Thank you for submitting your manuscript entitled “Psychological distress among women with abnormal cervical cancer screening result in Poland: Validity and reliability of the Cervical Dysplasia Distress Questionnaire (CDDQ)”

We have now received comments from two reviewers. Please revise your manuscript by carefully addressing each of the reviewers' comments.

For every point raised, please provide a detailed, point-by-point response indicating how the manuscript has been revised or, where appropriate, explaining the rationale if a suggested change was not made.

We look forward to receiving your revised manuscript.

Kind regards,

Kazunori Nagasaka

[Note: HTML markup is below. Please do not edit.]

Reviewers' comments:

Reviewer's Responses to Questions

**Comments to the Author**

1. Is the manuscript technically sound, and do the data support the conclusions?

Reviewer #1: Yes

Reviewer #2: Partly

**********

2. Has the statistical analysis been performed appropriately and rigorously?

Reviewer #1: Yes

Reviewer #2: Yes

**********

3. Have the authors made all data underlying the findings in their manuscript fully available?

The PLOS Data policy

Reviewer #1: Yes

Reviewer #2: No

**********

4. Is the manuscript presented in an intelligible fashion and written in standard English?

Reviewer #1: Yes

Reviewer #2: Yes

**********

Reviewer #1: This manuscript presents the translation, cultural adaptation, and psychometric validation of the Polish version of the Cervical Dysplasia Distress Questionnaire (CDDQ-PL). The topic is clinically relevant because psychological distress after abnormal cervical cancer screening results may influence adherence to diagnostic follow-up. The study is generally well designed, the statistical methodology is appropriate, and the manuscript contributes a useful validated instrument for Polish clinical practice. The psychometric evaluation is comprehensive and includes translation procedures, confirmatory factor analysis, internal consistency, convergent validity, and discriminant validity.

Test-retest reliability is missing. The manuscript evaluates internal consistency but does not assess temporal stability. Although the authors acknowledge this limitation, test-retest reliability is considered one of the fundamental psychometric properties of a newly validated instrument. The absence of test-retest analysis limits conclusions regarding measurement stability. The limitation should be emphasized more clearly in both the Discussion and Conclusions.

The manuscript evaluates construct validity, convergent validity and discriminant validity. However, criterion validity (predictive or known-group validity) was not assessed. Although the authors explain that predictive validity was beyond the scope of the study, they should discuss more explicitly why this property was omitted and how future studies could address it.

Participants were recruited from a single tertiary colposcopy center using convenience sampling. This introduces selection bias. Women attending a university referral center may differ substantially from those screened in primary care. The Discussion should better explain how this may influence generalizability.

Most participants had higher education and lived in large cities. This may not adequately represent the entire Polish screening population. The authors should discuss possible socioeconomic effects on distress perception and questionnaire performance.

The manuscript states that only approximately one-third of participants completed the SF-36 because it was introduced later during recruitment. This should be discussed as a methodological limitation because convergent/discriminant analyses were performed on only 95 women, reduced statistical power may affect validity estimates and potential selection bias cannot be excluded.

Overall, the statistical methodology is appropriate. The manuscript states that model fit indices met acceptable criteria. The manuscript should explicitly report accepted thresholds such as CFI >0.90 (or >0.95), RMSEA <0.08, SRMR <0.08 and explain why the observed values indicate good model fit. The authors report noticeable floor effects for items 2, 7 and 8. A quantitative definition should be provided.

The translation followed Brislin's model appropriately. However, the authors should clarify whether cognitive debriefing or pilot interviews were performed with Polish patients before the final version. Such testing is recommended in many cross-cultural adaptation guidelines. The manuscript mentions expert evaluation. Please specify number of experts, professional backgrounds and whether any quantitative content validity index (CVI) was calculated.

The Discussion could better explain how the questionnaire might be incorporated into routine cervical screening. For example, identifying women requiring psychological counselling, predicting non-adherence and improving communication before colposcopy.

I would recommend a slightly clearer title, Psychological distress among women with abnormal cervical cancer screening results in Poland: Validation of the Polish version of the Cervical Dysplasia Distress Questionnaire (CDDQ-PL)

Minor language editing is recommended.

Reviewer #2: Keywords could be diversified; please add other MeSH related keywords.

Please refrain from AI-generated phrases.

The introduction requires one clear aim sentence; as the tool was tranlated into Polish, please also add the term "translation" as well.

"Exclusion criteria included age <18 years and inability to understand Polish." Exclusion criteria should not be given as the opposites of the inclusion criteria.

"the Polish version was back-translated into English by an independent bilingual translator fluent in both Polish and English." "The translated version was additionally evaluated by healthcare professionals " The presentation of the translation and cross-cultural adaptation process needs particular attention. Please take recent recommendations PMID: 39716865 and revise this section accordingly. Details of translators (e.g., their background) should be provided, as well as the details of pilot testing (e.g., number of participants).

Exclusion of patients with incomplete questionnaires is questionable; what if the patients did not complete the questionnaires due to the potential deficiencies in translation/cross-cultural adaptation?

Each software should be given along with manufacturer and country of origin.

IQR is one single value.

Are Table 4 and 7 needed?

"Construct validity was further evaluated through correlation analyses." Whisch correlation analyses were used?

Some of the correlation coefficients are below 0.300.

The limitations paragraph could be expanded.

Does each author meet the first two criteria of ICMJE?

The reference list could be updated and diversified.

Future insights could be added.

**********

what does this mean?). If published, this will include your full peer review and any attached files.

If you choose “no”, your identity will remain anonymous but your review may still be made public.

**Do you want your identity to be public for this peer review?**  For information about this choice, including consent withdrawal, please see our Privacy Policy

Reviewer #1: **Yes:**  Ilker ILHANLI

Reviewer #2: No

**********

[NOTE: If reviewer comments were submitted as an attachment file, they will be attached to this email and accessible via the submission site. Please log into your account, locate the manuscript record, and check for the action link "View Attachments". If this link does not appear, there are no attachment files.]

To ensure your figures meet our technical requirements, please review our figure guidelines: https://journals.plos.org/plosone/s/figures

You may also use PLOS’s free figure tool, NAAS, to help you prepare publication quality figures: https://journals.plos.org/plosone/s/figures#loc-tools-for-figure-preparation.

NAAS will assess whether your figures meet our technical requirements by comparing each figure against our figure specifications.

# Author comment: Author response to Decision Letter 1

**DOI:** 10.1371/journal.pone.0360240.r002

26 Aug 2026

Journal Requirements

Comment 1. Please ensure that your manuscript meets PLOS ONE's style requirements, including those for file naming.

Response: The manuscript and accompanying files have been revised.

Comment 2. Thank you for stating the following in the Acknowledgments Section of your manuscript: “The article processing charge was funded by internal funds of the Jagiellonian University Medical College. The authors would like to thank all colleagues working at the colposcopy unit of the University Hospital in Kraków for their assistance in the dissemination of questionnaires and support during the study.”

We note that you have provided funding information that is not currently declared in your Funding Statement. However, funding information should not appear in the Acknowledgments section or other areas of your manuscript. We will only publish funding information present in the Funding Statement section of the online submission form.

Please remove any funding-related text from the manuscript and let us know how you would like to update your Funding Statement. Currently, your Funding Statement reads as follows:

“The author(s) received no specific funding for this work.”

Please include your amended statements within your cover letter; we will change the online submission form on your behalf.

Response: We have removed the sentence concerning the article processing charge from the Acknowledgments section. The study itself received no specific research funding; however, the article processing charge was covered by internal funds of the Jagiellonian University Medical College. We therefore request that the Funding Statement in the online submission form be amended as indicated below.

Update of Funding statement: The article processing charge was funded by internal funds of the Jagiellonian University Medical College.

Comment 3. In the online submission form you indicate that your data is not available for proprietary reasons and have provided a contact point for accessing this data. Please note that your current contact point is a co-author on this manuscript. According to our Data Policy, the contact point must not be an author on the manuscript and must be an institutional contact, ideally not an individual. Please revise your data statement to a non-author institutional point of contact, such as a data access or ethics committee, and send this to us via return email. Please also include contact information for the third party organization, and please include the full citation of where the data can be found.

Response: The authors have decided to make the dataset underlying the study publicly available. The data have been deposited in Kaggle and can be accessed without contacting an author or institutional representative at https://doi.org/10.34740/kaggle/dsv/19205304. We therefore request that the Data Availability Statement in the online submission form be replaced with the

The dataset underlying the findings of this study is publicly available: https://doi.org/10.34740/kaggle/dsv/19205304

Comment 4. Your ethics statement should only appear in the Methods section of your manuscript. If your ethics statement is written in any section besides the Methods, please move it to the Methods section and delete it from any other section. Please ensure that your ethics statement is included in your manuscript, as the ethics statement entered into the online submission form will not be published alongside your manuscript.

Response: The ethics statement has been moved to the Methods section

Comment 5. Please remove your figures from within your manuscript file, leaving only the individual TIFF/EPS image files, uploaded separately. These will be automatically included in the reviewers’ PDF.

Response: The figures have been removed from the manuscript file.

Comment 6. Please include captions for your Supporting Information files at the end of your manuscript, and update any in-text citations to match accordingly. Please see our Supporting Information guidelines for more information: http://journals.plos.org/plosone/s/supporting-information.

Response: Supporting Information files are included. The study dataset is publicly available through the external Kaggle repository cited in the Data Availability Statement and is not submitted as Supporting Information.

Comment 7. If the reviewer comments include a recommendation to cite specific previously published works, please review and evaluate these publications to determine whether they are relevant and should be cited. There is no requirement to cite these works unless the editor has indicated otherwise.

Response: The recommended publications were reviewed and evaluated for their relevance to the study.

Comment 8. Please review your reference list to ensure that it is complete and correct. If you have cited papers that have been retracted, please include the rationale for doing so in the manuscript text, or remove these references and replace them with relevant current references. Any changes to the reference list should be mentioned in the rebuttal letter that accompanies your revised manuscript. If you need to cite a retracted article, indicate the article’s retracted status in the References list and also include a citation and full reference for the retraction notice.

Response: The reference list has been carefully reviewed.

Reviewer #1

Comment 1: Test-retest reliability is missing. The manuscript evaluates internal consistency but does not assess temporal stability. Although the authors acknowledge this limitation, test-retest reliability is considered one of the fundamental psychometric properties of a newly validated instrument. The absence of test-retest analysis limits conclusions regarding measurement stability. The limitation should be emphasized more clearly in both the Discussion and Conclusions.

Response to comment 1: We agree that internal consistency alone does not establish the temporal stability of an instrument and that test–retest reliability is an essential component of a comprehensive psychometric evaluation. Because the present study employed a cross-sectional design and each participant completed the CDDQ-PL only once, test–retest reliability could not be assessed retrospectively. We have therefore strengthened the relevant limitation in the Discussion and explicitly stated that the absence of repeated measurements prevents conclusions regarding the temporal stability and reproducibility of CDDQ-PL scores. We have also revised the Conclusions to avoid implying that all aspects of reliability have been established. The revised text now describes the findings as evidence of validity and good internal consistency, while emphasizing that prospective studies using an appropriate retest interval are required before the temporal stability of the CDDQ-PL can be confirmed.

Comment 2: The manuscript evaluates construct validity, convergent validity and discriminant validity. However, criterion validity (predictive or known-group validity) was not assessed. Although the authors explain that predictive validity was beyond the scope of the study, they should discuss more explicitly why this property was omitted and how future studies could address it.

Response to comment 2: We agree that the absence of criterion-related validity assessment should be explained more explicitly. Criterion validity was not evaluated because no validated Polish condition-specific instrument or accepted diagnostic gold standard was available against which CDDQ-PL scores could be compared. Furthermore, the cross-sectional design did not include longitudinal clinical or behavioral outcomes, such as attendance at recommended follow-up examinations or engagement with treatment, and therefore did not permit assessment of predictive validity. Known-group comparisons were also not planned because the study did not prospectively define sufficiently distinct clinical groups expected to differ in distress.

Comment 3: Participants were recruited from a single tertiary colposcopy center using convenience sampling. This introduces selection bias. Women attending a university referral center may differ substantially from those screened in primary care. The Discussion should better explain how this may influence generalizability.

Response to comment 3: We agree that recruitment through convenience sampling at a single tertiary colposcopy center may have introduced selection bias and limits the generalizability of our findings. Women referred to a university-based specialist center may differ from women undergoing routine screening in primary care with respect to the severity or complexity of their clinical findings, sociodemographic characteristics, health literacy, access to healthcare, previous screening experiences, and levels of distress. In addition, women who attended the referral appointment and agreed to participate may have differed from those who did not attend or declined participation.

Comment 4: Most participants had higher education and lived in large cities. This may not adequately represent the entire Polish screening population. The authors should discuss possible socioeconomic effects on distress perception and questionnaire performance.

Response to comment 4: We agree that the predominance of participants with higher education and residence in large cities limits the sociodemographic representativeness of the sample. Educational level, health literacy, socioeconomic circumstances, and access to healthcare may affect how women understand abnormal screening results, perceive and report distress, and interpret questionnaire items. Consequently, the distribution of CDDQ-PL scores and some of its psychometric properties may differ in women with lower educational attainment, lower health literacy, financial or healthcare-access barriers, or residence in rural and underserved areas.

Comment 5: The manuscript states that only approximately one-third of participants completed the SF-36 because it was introduced later during recruitment. This should be discussed as a methodological limitation because convergent/discriminant analyses were performed on only 95 women, reduced statistical power may affect validity estimates and potential selection bias cannot be excluded.

Response to comment 5: We agree that the smaller number of participants who completed the SF-36 affects the interpretation of the analyses involving quality-of-life domains. Because the SF-36 was introduced later in the recruitment period, these analyses were based on 95 participants rather than the full sample of 285. This reduced sample size lowered statistical power, increased the uncertainty of the correlation estimates, and limited the ability to detect weaker associations. Furthermore, because SF-36 availability was determined by the recruitment period, potential differences between participants recruited before and after its introduction cannot be excluded, introducing a possible source of selection bias. We have added this limitation to the Discussion and have clarified that the findings based on correlations between the CDDQ-PL and SF-36 should be considered preliminary and require confirmation in larger samples in which both instruments are administered to all participants.

Comment 6: Overall, the statistical methodology is appropriate. The manuscript states that model fit indices met acceptable criteria. The manuscript should explicitly report accepted thresholds such as CFI >0.90 (or >0.95), RMSEA <0.08, SRMR <0.08 and explain why the observed values indicate good model fit. The authors report noticeable floor effects for items 2, 7 and 8. A quantitative definition should be provided.

Response to comment 6: We have revised the Statistical methods and Results sections to report the model-fit criteria and observed values explicitly.

Comment 7: The translation followed Brislin's model appropriately. However, the authors should clarify whether cognitive debriefing or pilot interviews were performed with Polish patients before the final version. Such testing is recommended in many cross-cultural adaptation guidelines. The manuscript mentions expert evaluation. Please specify number of experts, professional backgrounds and whether any quantitative content validity index (CVI) was calculated.

Response to comment 7: We have expanded the description of the translation and cultural-adaptation process.

Comment 8: The Discussion could better explain how the questionnaire might be incorporated into routine cervical screening. For example, identifying women requiring psychological counselling, predicting non-adherence and improving communication before colposcopy.

Response to comment 8: We have expanded the Discussion to describe potential applications of the CDDQ-PL within the cervical screening pathway. The questionnaire could be administered after notification of an abnormal screening result or before colposcopy to identify specific concerns and facilitate communication between patients and healthcare professionals. Elevated scores or responses indicating marked distress could prompt further clinical assessment and, where appropriate, referral for psychological counselling or additional educational support.

Comment 9: I would recommend a slightly clearer title, Psychological distress among women with abnormal cervical cancer screening results in Poland: Validation of the Polish version of the Cervical Dysplasia Distress Questionnaire (CDDQ-PL)

Response to comment 9: We thank the Reviewer for this helpful suggestion. We agree that the proposed title more clearly identifies the study as a validation of the Polish version of the instrument and introduces the CDDQ-PL abbreviation consistently. We have revised the manuscript title accordingly.

Comment 10: Minor language editing is recommended.

Response to comment 10: The manuscript has been carefully reviewed and edited.

Reviewer #2

Comment 1: Keywords could be diversified; please add other MeSH related keywords.

Response to comment 1: We have revised and diversified the keywords using relevant MeSH.

Comment 2: Please refrain from AI-generated phrases.

Response to comment 2: The manuscript has been thoroughly revised by the authors to remove formulaic, repetitive, or overly generalized wording and to ensure that the language accurately reflects the study methods, results, and interpretations. All revised passages were critically reviewed and approved by the authors, who take full responsibility for the content of the manuscript.

Comment 3: The introduction requires one clear aim sentence; as the tool was tranlated into Polish, please also add the term "translation" as well.

Response to comment 3: We have revised the final paragraph of the Introduction to provide one explicit study aim. The revised sentence now states that the study included translation and cultural adaptation of the CDDQ into Polish, as well as evaluation of its psychometric properties.

Comment 4: "Exclusion criteria included age <18 years and inability to understand Polish." Exclusion criteria should not be given as the opposites of the inclusion criteria.

Response to comment 4: We agree that the stated exclusion criteria merely repeated the inclusion criteria in reverse. We have therefore removed the redundant sentence and retained a single, clear description of participant eligibility.

Comment 5: "the Polish version was back-translated into English by an independent bilingual translator fluent in both Polish and English." "The translated version was additionally evaluated by healthcare professionals " The presentation of the translation and cross-cultural adaptation process needs particular attention. Please take recent recommendations PMID: 39716865 and revise this section accordingly. Details of translators (e.g., their background) should be provided, as well as the details of pilot testing (e.g., number of participants).

Response to comment 5: We have expanded the description of the translation and cultural-adaptation process.

Comment 6: Exclusion of patients with incomplete questionnaires is questionable; what if the patients did not complete the questionnaires due to the potential deficiencies in translation/cross-cultural adaptation?

Response to comment 6: Before completing the questionnaire, all participants were informed that they could ask questions or request clarification at any time during its completion. After returning the questionnaire, each participant was also offered an opportunity to discuss it with the physician and was specifically asked whether any item had been unclear or difficult to understand. The feedback obtained through this procedure did not indicate recurring problems with the wording, meaning, or cultural relevance of the Polish CDDQ items.

Comment 7: Each software should be given along with manufacturer and country of origin.

Response to comment 7: The Statistical methods section has been revised to provide the data of the software used in the analyses.

Comment 8: IQR is one single value.

Response to comment 8: We thank the Reviewer for this correction.

Comment 9: Are Table 4 and 7 needed?

Response to comment 9: The authors would prefer to retain both tables because they provide a clear and readily accessible presentation of relevant results. Table 4 presents the complete descriptive distribution of anxiety scores before and after the visit, including means, standard deviations, medians, quartiles, and ranges. Table 7 enables a direct comparison of internal-consistency coefficients across the CDDQ-PL subscales. We believe that retaining these tables improves the transparency and readability of the results.

Comment 10: "Construct validity was further evaluated through correlation analyses." Which correlation analyses were used?

Response to comment 10: Spearman’s rank-order correlation analysis was used. Specifically, Spearman’s correlation coefficients were calculated between the CDDQ-PL total and subscale scores and anxiety scores before and after the visit to assess convergent validity. Correlations between the CDDQ-PL scores and individual SF-36 domains were also examined to assess the expected relationships with health-related quality of life. The Statistical methods section has been revised accordingly.

Comment 11: Some of the correlation coefficients are below 0.300.

Response to comment 11: We agree that several statistically significant correlations below 0.300 represent weak rather than moderate associations. We have revised the Results.

Comment 12: The limitations paragraph could be expanded.

Response to comment 12: The Limitations section has been expanded.

Comment 13: Does each author meet the first two criteria of ICMJE?

Response to comment 13: The Authors’ Contributions statement has been revised to specify each author’s substantial contribution

Comment 14: The reference list could be updated and diversified.

Response to comment 14: Done.

Comment 15: Future insights could be added.

Response to comment 15: The Discussion has been revised to include directions for future research.

Attachment Submitted filename: Response to Reviewers.docx

# Peer review: Decision Letter 1

**DOI:** 10.1371/journal.pone.0360240.r003

**Authors:** Kazunori Nagasaka

24 Sep 2026

Psychological distress among women with abnormal cervical cancer screening results in Poland: Validation of the Polish version of the Cervical Dysplasia Distress Questionnaire (CDDQ-PL)

PONE-D-26-27960R1

Dear Dr. Trojnarska,

We’re pleased to inform you that your manuscript has been judged scientifically suitable for publication and will be formally accepted for publication once it meets all outstanding technical requirements.

Within one week, you’ll receive an e-mail detailing the required amendments. When these have been addressed, you’ll receive a formal acceptance letter and your manuscript will be scheduled for publication.

An invoice will be generated when your article is formally accepted. Please note, if your institution has a publishing partnership with PLOS and your article meets the relevant criteria, all or part of your publication costs will be covered. Please make sure your user information is up-to-date by logging into Editorial Manager at Editorial Manager® and clicking the ‘Update My Information' link at the top of the page. For questions related to billing, please contact billing support.

If your institution or institutions have a press office, please notify them about your upcoming paper to help maximize its impact. If they’ll be preparing press materials, please inform our press team as soon as possible -- no later than 48 hours after receiving the formal acceptance. Your manuscript will remain under strict press embargo until 2 pm Eastern Time on the date of publication. For more information, please contact onepress@plos.org.

Kind regards,

Kazunori Nagasaka

Academic Editor

PLOS One

Additional Editor Comments (optional):

Dear Authors,

Thank you for submitting your article, entitled ”Psychological distress among women with abnormal cervical cancer screening results in Poland: Validation of the Polish version of the Cervical Dysplasia Distress Questionnaire (CDDQ-PL)”.

After careful consideration, we decided to accept your manuscript for publication.

Congulattration, and thank you for your valuable contribution to the journal.

Kind regards,

Kazunori Nagasaka

Reviewers' comments:

Reviewer's Responses to Questions

**Comments to the Author**

Reviewer #1: All comments have been addressed

Reviewer #2: All comments have been addressed

**********

2. Is the manuscript technically sound, and do the data support the conclusions?

Reviewer #1: Yes

Reviewer #2: Yes

**********

3. Has the statistical analysis been performed appropriately and rigorously?

Reviewer #1: Yes

Reviewer #2: Yes

**********

4. Have the authors made all data underlying the findings in their manuscript fully available?

The PLOS Data policy

Reviewer #1: Yes

Reviewer #2: No

**********

5. Is the manuscript presented in an intelligible fashion and written in standard English?

Reviewer #1: Yes

Reviewer #2: Yes

**********

Reviewer #1: Thanks to authors for revision and their efforts. This manuscript presents the translation, cultural adaptation, and psychometric validation of the Polish version of the Cervical Dysplasia Distress Questionnaire (CDDQ-PL). The topic is clinically relevant because psychological distress after abnormal cervical cancer screening results may influence adherence to diagnostic follow-up. The study is generally well designed, the statistical methodology is appropriate, and the manuscript contributes a useful validated instrument for Polish clinical practice. The psychometric evaluation is comprehensive and includes translation procedures, confirmatory factor analysis, internal consistency, convergent validity, and discriminant validity.

Reviewer #2: Thank you for the corrections. The manuscript has improved substantially following revisions. Kind regards

**********

what does this mean?). If published, this will include your full peer review and any attached files.

If you choose “no”, your identity will remain anonymous but your review may still be made public.

**Do you want your identity to be public for this peer review?**  For information about this choice, including consent withdrawal, please see our Privacy Policy

Reviewer #1: **Yes:**  Ilker ILHANLI

Reviewer #2: No

**********

# Editor report: Acceptance letter

**DOI:** 10.1371/journal.pone.0360240.r004

**Authors:** Kazunori Nagasaka

PONE-D-26-27960R1

PLOS One

Dear Dr. Trojnarska,

I'm pleased to inform you that your manuscript has been deemed suitable for publication in PLOS One. Congratulations! Your manuscript is now being handed over to our production team.

At this stage, our production department will prepare your paper for publication. This includes ensuring the following:

* All references, tables, and figures are properly cited

* All relevant supporting information is included in the manuscript submission,

* There are no issues that prevent the paper from being properly typeset

You will receive further instructions from the production team, including instructions on how to review your proof when it is ready. Please keep in mind that we are working through a large volume of accepted articles, so please give us a few days to review your paper and let you know the next and final steps.

Lastly, if your institution or institutions have a press office, please let them know about your upcoming paper now to help maximize its impact. If they'll be preparing press materials, please inform our press team within the next 48 hours. Your manuscript will remain under strict press embargo until 2 pm Eastern Time on the date of publication. For more information, please contact onepress@plos.org.

You will receive an invoice from PLOS for your publication fee after your manuscript has reached the completed accept phase. If you receive an email requesting payment before acceptance or for any other service, this may be a phishing scheme. Learn how to identify phishing emails and protect your accounts at https://explore.plos.org/phishing.

If we can help with anything else, please email us at customercare@plos.org.

Thank you for submitting your work to PLOS One and supporting open access.

Kind regards,

PLOS One Editorial Office Staff

on behalf of

Professor Kazunori Nagasaka

Academic Editor

PLOS One
